# Identification of galactofuranose antigens such as galactomannoproteins and fungal-type galactomannan from the yellow *koji* fungus (*Aspergillus oryzae*)

**DOI:** 10.3389/fmicb.2023.1110996

**Published:** 2023-02-06

**Authors:** Chihiro Kadooka, Yutaka Tanaka, Daisuke Hira, Jun-ichi Maruyama, Masatoshi Goto, Takuji Oka

**Affiliations:** ^1^Department of Biotechnology and Life Sciences, Faculty of Biotechnology and Life Sciences, Sojo University, Kumamoto, Japan; ^2^Division of Infection and Host Defense, Tohoku Medical and Pharmaceutical University, Sendai, Japan; ^3^Department of Biotechnology, The University of Tokyo, Tokyo, Japan; ^4^Collaborative Research Institute for Innovative Microbiology, The University of Tokyo, Tokyo, Japan; ^5^Department of Applied Biochemistry and Food Science, Faculty of Agriculture, Saga University, Saga, Japan

**Keywords:** UDP-galactopyranose mutase (UGM), fungal-type galactomannan, galactofuranose, cell wall, *Aspergillus oryzae*

## Abstract

Filamentous fungi belonging to the genus *Aspergillus* are known to possess galactomannan in their cell walls. Galactomannan is highly antigenic to humans and has been reported to be involved in the pathogenicity of pathogenic filamentous fungi, such as *A. fumigatus*, and in immune responses. In this study, we aimed to confirm the presence of D-galactofuranose-containing glycans and to clarify the biosynthesis of D-galactofuranose-containing glycans in *Aspergillus oryzae*, a yellow *koji* fungus. We found that the galactofuranose antigen is also present in *A. oryzae*. Deletion of *ugmA*, which encodes UDP-galactopyranose mutase in *A. oryzae*, suppressed mycelial elongation, suggesting that D-galactofuranose-containing glycans play an important role in cell wall integrity in *A. oryzae*. Proton nuclear magnetic resonance spectrometry revealed that the galactofuranose-containing sugar chain was deficient and that core mannan backbone structures were present in Δ*ugmA A. oryzae*, indicating the presence of fungal-type galactomannan in the cell wall fraction of *A. oryzae*. The findings of this study provide new insights into the cell wall structure of *A. oryzae*, which is essential for the production of fermented foods in Japan.

## 1. Introduction

Galactomannan (GM) is a polysaccharide composed of D-mannose (Man) and D-galactofuranose (Gal*f*). GM functions as a component of the cell wall in filamentous fungi (Tefsen et al., 2012; Gow et al., 2017; Oka, 2018). The detailed structure of GM has been elucidated in *Aspergillus fumigatus*, a major pathogenic fungus that causes invasive pulmonary aspergillosis (Latgé et al., 1994; Kudoh et al., 2015). *A. fumigatus* possesses two types of GM: O-mannose–type GM (OMGM) and fungal-type GM (FTGM) (Kudoh et al., 2015; Katafuchi et al., 2017; Oka, 2018). OMGM is a galactomannoprotein consisting of an α-(1,2)-mannosyl chain attached to the hydroxyl group of a serine and/or threonine residue in the protein and a galactofuran side chain comprising a β-(1,5)−/β-(1,6)-galactofuranosyl chain attached to a Man residue (Oka et al., 2004; Goto et al., 2009; Kudoh et al., 2015). FTGM has a linear α-mannan backbone consisting of 9–10 α-(1,2)-mannotetraose units linked by α-(1,6) bonds (Latgé et al., 1994; Kudoh et al., 2015; Kadooka et al., 2022a), and its galactofuran side chains are β-(1,2)-, β-(1,3)-, and/or β-(1,6)-linked to this α-core-mannan (Latgé et al., 1994; Kudoh et al., 2015). FTGM is biosynthesized in the Golgi apparatus and is presumed to be transported to the cell surface *via* the glycosylphosphatidylinositol (GPI) anchor as a carrier molecule (Costachel et al., 2005; Fontaine and Latgé, 2020). By consolidating the findings from recent studies, it can be speculated that FTGMs that are transported to the cell surface can covalently bind to β-glucan, further solidifying the cell wall structure, and some may be released into extracellular compartments, such as the culture supernatant (Muszkieta et al., 2019; Fontaine and Latgé, 2020; Vogt et al., 2020).

Gal*f* is highly antigenic to humans and is commonly detected in the blood of patients with invasive aspergillosis (Stynen et al., 1992). Therefore, Gal*f* is considered a virulence factor for the pathogenicity of *A. fumigatus*. Single-gene disruption of *glfA*, which encodes UDP-galactopyranose mutase, the primary enzyme involved in the biosynthesis of Gal*f*-containing polysaccharides, was reported to induce temperature sensitivity and to significantly reduce the pathogenicity of *A. fumigatus* to mice (Bakker et al., 2005; Schmalhorst et al., 2008). Subsequently, *glfA* (renamed *Afugm1*) was analyzed in different strains of *A. fumigatus*, and the loss of Gal*f* antigens was found to have little effect on pathogenicity (Lamarre et al., 2009). Although the relevance of Gal*f* antigens to pathogenicity is unclear, their antigenic properties suggest that they are involved in certain immune responses in host cells.

The yellow *koji* fungus, *Aspergillus oryzae*, has been listed as “generally recognized as safe” by the US Food and Drug Administration (Machida, 2002). *A. oryzae* is a filamentous fungus used in the production of sake, miso, and soy sauce in Japan and in many fermentation industries owing to its safety (Kitamoto, 2015; Ichishima, 2016; Gomi, 2019; Kitagaki, 2021). Because *A. oryzae*, like *A. fumigatus*, is a filamentous fungus belonging to the subphylum *Pezizomycotina*, it may contain Gal*f*-containing polysaccharides as cell wall components. In a previous study, Gal*f*-containing glycan structures were detected in the cell wall alkali-soluble fraction of *A. oryzae* from which O-linked glycans were removed and β-(1,2)-Gal*f* was added to the N-glycan outer chain structures (Nakajima and Ichishima, 1994). Furthermore, the genome of *A. oryzae* has been reported to contain the genes *AougmA* and *AougmB*, which are presumed to encode UDP-galactopyranose mutase (Damveld et al., 2008). Because *A. oryzae* is used in food production, it is important to analyze the Gal*f*-containing glycan structure in detail to ensure its safety.

Thus, this study aimed to clarify the structure of Gal*f*-containing polysaccharides in *A. oryzae*. Our immunoblot analysis using an anti-Gal*f* antibody revealed the presence of a small amount of galactomannoprotein, a Gal*f* antigen, in *A. oryzae*. We also found that *A. oryzae ugmA* encodes UDP-galactopyranose mutase and that Gal*f*-containing polysaccharides are important for normal mycelial elongation in *A. oryzae*. In addition, proton nuclear magnetic resonance (^1^H-NMR) spectrometry revealed the presence of an FTGM-like structure in *A. oryzae*.

## 2. Materials and methods

### 2.1. Strains and growth conditions

The *Aspergillus* strains used in this study are listed in Supplementary Table S1. The strains were grown on minimal medium (MM) containing 1% w/v glucose, 10 mM sodium glutamate, 0.052% w/v KCl, 0.052% w/v MgSO_4_·7H_2_O, and 0.152% w/v KH_2_PO_4_, plus Hunter’s trace elements (pH 6.5). To cultivate *A. oryzae* NSPlD1 (Maruyama and Kitamoto, 2008), 1.5 g/l methionine, 1.22 g/l uracil, and 1.21 g/l uridine were added to MM. To cultivate the *Aspergillus nidulans* strains, 1 mg/l biotin was added to MM.

### 2.2. Construction of the *ugmA*-disrupted strain

*ugmA* was disrupted in *A. oryzae* NSPlD1 by inserting *AnpyrG*. A gene replacement cassette encompassing the homology arms at the 5′ and 3′ ends of *ugmA* was amplified by recombinant polymerase chain reaction (PCR) using *A. oryzae* RIB40 genomic DNA as the template and the primer pairs ugmA-1/ugmA-2 and ugmA-3/ugmA-4, respectively (Supplementary Table S2). The *A. nidulans pyrG* (*AnpyrG*) marker was amplified by recombinant PCR using pHSG396-AnpyrG (Kadooka et al., 2022b) as the template and the primer pair pHSG396-F/pHSG396-R. The resultant DNA fragment, amplified using the primers ugmA-1 and ugmA-4, was used to transform *A. oryzae* NSPlD1, yielding the Δ*ugmA* strain. MM agar plates without uracil and uridine were used to select the transformants. The introduction of *AnpyrG* into each gene locus was confirmed by PCR using the primer pair ugmA-F/ugmA-R (Supplementary Figure S1).

### 2.3. Construction of pPTR-II-ugmA

*ugmA* that included 1.5 kbp upstream of *ugmA* was amplified by PCR using *A. oryzae* RIB40 genomic DNA as the template and the primer pair pPTR-II-ugmA-IF-F/pPTR-II-ugmA-IF-R. The amplified fragment was inserted into the *Sma*I site of pPTR-II using the In-Fusion HD Cloning Kit (Takara, Kusatsu, Shiga, Japan) to yield pPTR-II-ugmA.

### 2.4. Preparation of the GM fraction

Total GM (FTGM and galactomannoproteins) from *A. oryzae* was prepared as previously described (Katafuchi et al., 2017). Briefly, the hot-water-soluble extract from cells was fractionated using cetyl trimethyl ammonium bromide. The resultant fraction was precipitated at pH 9.0 with NaOH in the presence of borate and resolved in distilled water as the total GM fraction. A β-elimination reaction was performed to remove O-glycans from the galactomannoproteins under reducing alkali conditions (500 mM NaBH_4_ /100 mM NaOH, 10 ml, at 25°C for 24 h). After neutralization with 50% acetic acid, the samples were dialyzed overnight against distilled water. The purified samples were then lyophilized, resuspended in distilled water, and clarified using 0.45-μm-pore filters. The resultant samples were prepared as GM fractions.

### 2.5. ^1^H-NMR spectroscopy

Samples for ^1^H-NMR were exchanged twice in D_2_O with intervening lyophilization and then dissolved in D_2_O (99.97% atom ^2^H). The ^1^H-NMR spectra were recorded using a JNM-LA600 spectrometer (JEOL, Akishima, Tokyo, Japan) at 45°C. The proton chemical shifts were referenced relative to internal acetone at δ 2.225.

### 2.6. Immunoblotting

Immunoblotting was performed as previously described (Komachi et al., 2013). The EB-A2 antibody of the Platelia *Aspergillus* enzyme immunoassay (Bio-Rad Laboratories, Hercules, CA, United States) was used at a dilution of 1:10 to detect β-Gal*f*.

### 2.7. Analysis of surface adhesion

Hyphal surface adhesion assay was performed as previously described with slight modifications (Lamarre et al., 2009; Alam et al., 2014). Briefly, 0.5-μm-diameter polystyrene beads (Sigma) were diluted to 1:100 in sterile phosphate-buffered saline (PBS). Mycelia were grown for 18 h at 30°C with shaking at 127 rpm in liquid potato dextrose medium, harvested into PBS-containing polystyrene beads for 1 h, and then washed five times with PBS. Mycelium images were acquired using a microscope equipped with a digital camera.

## 3. Results

### 3.1. Detection of Gal*f*-containing glycoprotein in *koji* fungi

**Figure 1:**
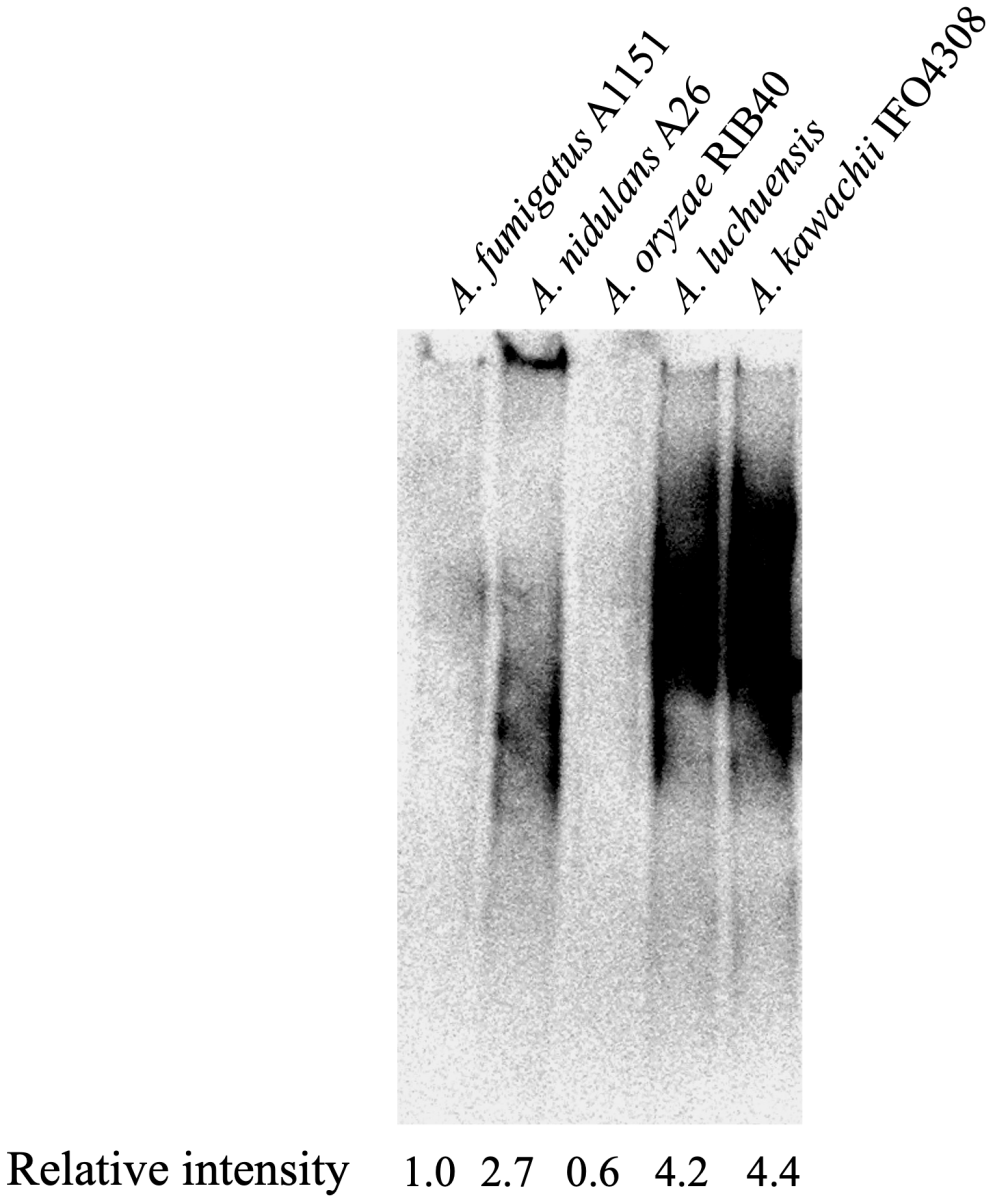
Immunoblot analysis of galactomannoproteins from *Aspergillus* spp. The presence of Gal*f*-containing glycoproteins was detected using EB-A2. Lanes 1–5: 20 μg of galactomannoproteins from *Aspergillus fumigatus* A1151 (Lane 1), *Aspergillus nidulans* A26 (Lane 2), *Aspergillus oryzae* RIB40 (Lane 3), *Aspergillus luchuensis* (Lane 4), and *Aspergillus kawachii* IFO4308 (Lane 5) were loaded. Densitometric quantification of immunoblotting bands was performed using ImageJ software (Schneider et al., 2012). The intensity ratios of EB-A2 for galactomannoproteins were calculated, and the ratio for *A. fumigatus* A1151 was normalized to 1.0.

To investigate the presence of Gal*f*-containing sugar chains in *A. oryzae*, *Aspergillus luchuensis* (*Aspergillus awamori* var. *kawachi*) and *A. luchuensis* mut. *Kawachii* (*A. kawachii*), galactomannoproteins were extracted from mycelia and subjected to immunoblotting to detect Gal*f*-containing glycoproteins using the anti-Gal*f* antibody EB-A2 (Oka et al., 2005). Smeared bands indicated the presence of Gal*f*-containing glycoproteins (Figure 1), which were thought to be mainly attributable to the O-glycans among the glycoproteins (Komachi et al., 2013). Densitometric quantification of the immunoblot bands was performed using ImageJ software (Schneider et al., 2012). The signal intensity ratios for EB-A2 were then calculated, and the ratio for *A. fumigatus* was normalized to 1.0. The ratio of EB-A2 intensity to Gal*f*-containing glycoprotein in *A. oryzae* was 0.6-fold less than that in *A. fumigatus* (Figure 1), suggesting the presence of few Gal*f*-containing glycoproteins in *A. oryzae* (Figure 1). Interestingly, the intensities of *A. luchuensis* and *A. kawachii* were more than four times higher than that of *A. fumigatus* (Figure 1). These data indicate that the quantities of Gal*f*-containing glycoproteins differ among different species of *koji* fungi.

### 3.2. Putative UDP-galactopyranose mutase in *koji* fungi

To investigate whether the genes encoding UDP-galactopyranose mutase are present in the genome of *koji* fungi, we performed an NCBI protein BLAST^1^ search using the amino acid sequence of *A. nidulans* UgmA (AN3112) as a query sequence. AO090012000855 of *A. oryzae* RIB40, RIB2604_01707610 of *A. luchuensis* RIB2604, and AKAW2_10730A of *A. kawachii* IFO4308 exhibited strong homology to *A. nidulans* UgmA (91.75, 91.18, and 91.35% identities, respectively).

**Figure 2:**
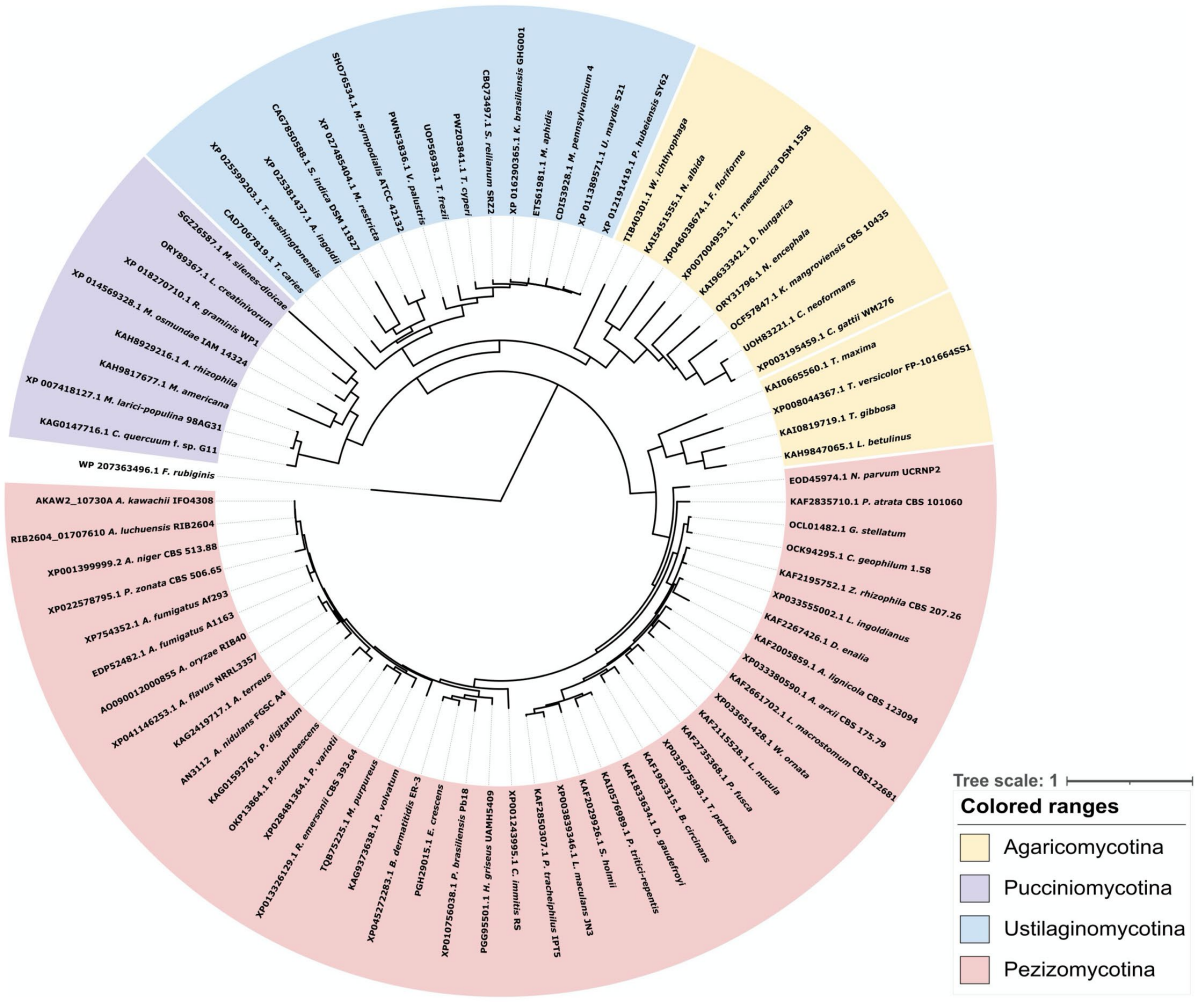
Phylogenetic analysis of UDP-galactopyranose mutase family proteins from bacteria, *Basidiomycota*, and *Pezizomycotina* species. Protein sequences were downloaded from NCBI. The phylogenetic tree was drawn using iTOL, and the alignment and phylogenetic tree inference were performed using MAFFT and RAxML, included in ETE v3. The UgmA homologous protein from the bacteria *Fibrella rubiginis*, presumably with a common ancestor, was used as the outgroup.

To clarify the diversity of GlfA/UgmA in detail, we performed a phylogenetic analysis of UDP-galactopyranose mutase conserved in fungi (Figure 2). The dataset for analysis was obtained by an NCBI protein BLAST search using the amino acid sequence of *A. nidulans* UgmA as a query sequence. The BLAST search identified the UDP-galactopyranose mutase of *A. oryzae*, which was named AoUgmA by Damveld et al. (2008). In the present study, AoUgmA is referred to as UgmA. Damveld et al. (2008) also revealed the presence of a putative UDP-galactopyranose mutase named AoUgmB in *A. oryzae*. However, as AoUgmB encodes only 168 amino acids, it probably does not function as a UDP-galactopyranose mutase because its protein size is too small. Therefore, AoUgmB was excluded from the present analysis. Phylogenetic analysis revealed that UgmA is widely distributed in the subphylum *Pezizomycotina* and the phylum *Basidiomycota*. In the phylum *Ascomycota*, *Saccharomycotina* and *Taphrinomycotina* do not carry UgmA. These results are consistent with previously reported findings and with the fact that galactofuranosyl chains are not present in *Saccharomycotina* and *Taphrinomycotina* (Tefsen et al., 2012). UgmA is present in *Agaricomycotina*, *Ustilaginomycotina*, and *Pucciniomycotina* in the phylum *Basidiomycota*, whereas it is absent in most *Agaricomycotina* in the phylum *Basidiomycota*. Functional analysis of UgmA in basidiomycetes is currently limited, and further functional analysis is warranted in future studies.

### 3.3. Phenotypic analysis of the *Aspergillus oryzae ugmA* disruptant

**Figure 3:**
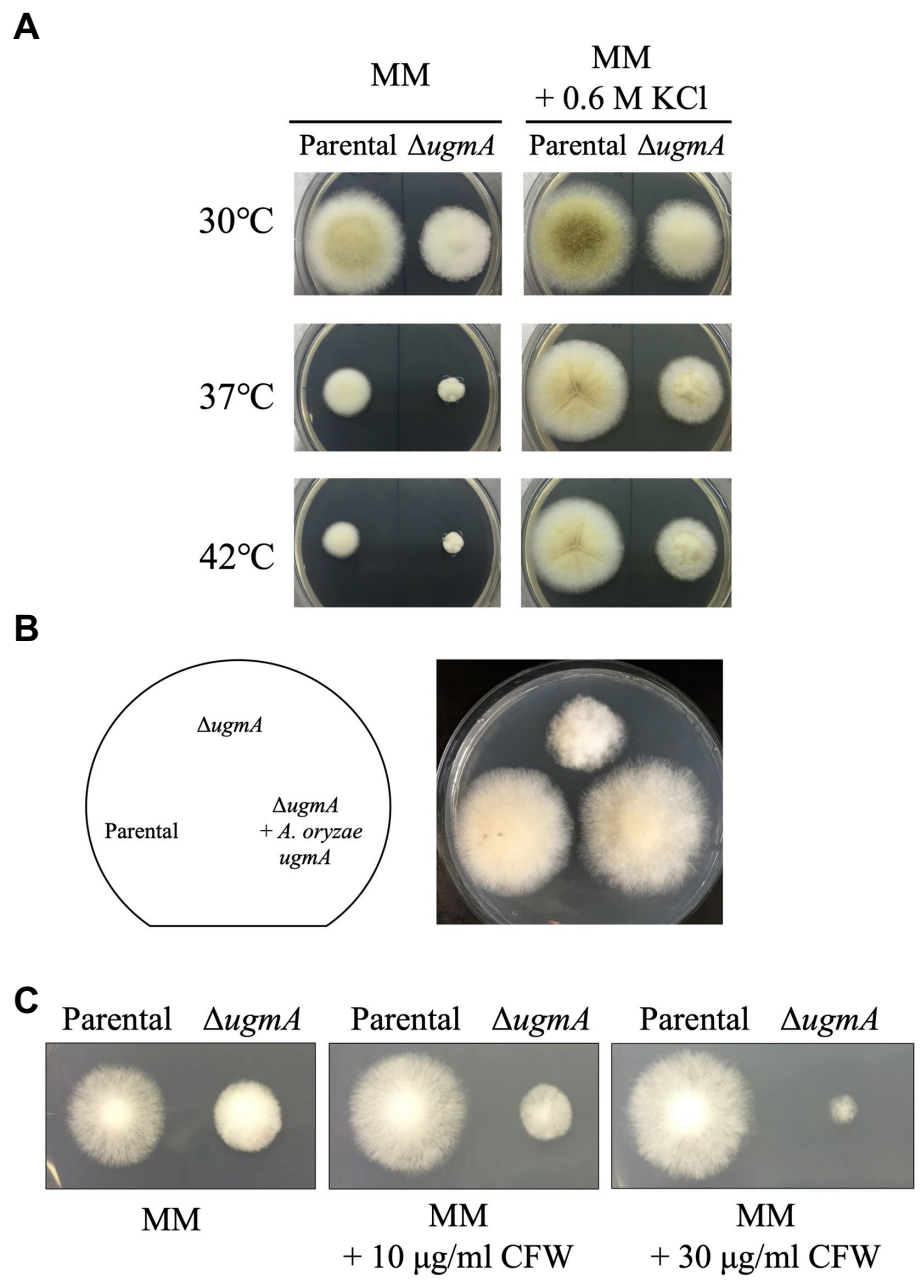
Phenotypic analysis of the Δ*ugmA* strain. **(A)** Colonial morphology of parental (NSPlD1) and Δ*ugmA* strains on minimal medium (MM) agar and MM agar supplemented with 0.6 M KCl after culture at 30°C, 37°C, or 42°C for 4 days. Agar medium was inoculated with 1.0 × 10^4^ conidiospores. **(B)** Colonial morphology of the parental, Δ*ugmA*, and Δ*ugmA* + *A. oryzae ugmA* strains after culture on MM agar for 4 days. Agar medium was inoculated with 1.0 × 10^4^ conidiospores. **(C)** Sensitivity to the cell wall stress inducer calcofluor white (CFW). Parental and Δ*ugmA* strains were grown on MM agar supplemented with 10 or 30 μg/ml CFW at 30°C for 3 days.

To investigate the physiological roles of *ugmA* in *A. oryzae*, we constructed a *ugmA* gene disruptant and observed the colonial morphology following culture at 30°C, 37°C, and 42°C for 5 days (Figure 3A). Δ*ugmA* had a smaller colony size than the parental strain, indicating the important role of UgmA in normal mycelial formation in *A. oryzae*. The growth of the parental and ∆*ugmA* strains was delayed at 42°C compared with that at 30°C on MM. The colony diameters of the parental strain were 34% smaller after culture for 4 days at 42°C compared with that at 30°C, while those of the ∆*ugmA* strain were 23% smaller after culture for 4 days at 42°C compared with that at 30°C. These results indicate that the ∆*ugmA* strain exhibits a temperature-sensitive phenotype (Figure 3A; Supplementary Figure S2). This temperature-sensitive phenotype tended to improve under high osmotic support conditions (Figure 3A), suggesting that the Δ*ugmA* strain exhibits temperature sensitivity attributable to the absence of some cell wall components. To confirm that the Δ*ugmA* phenotype was caused by the disruption of *ugmA* (Figure 3B), we constructed a *ugmA* complementation strain (Δ*ugmA* + *A. oryzae ugmA*) and observed the colonies (Figure 3B). The Δ*ugmA* + *A. oryzae ugmA* strain had a similar colony size and morphology as the parental strain on MM (Figure 3B). This result indicated that the abnormal phenotypes of Δ*ugmA* were truly attributable to *ugmA* disruption.

In addition, we investigated the drug sensitivity of Δ*ugmA*. In *A. nidulans* and *A. niger*, *ugmA*-disrupted strains were shown to be sensitive to higher concentrations of calcofluor white (CFW), a chitin-binding reagent (Damveld et al., 2008; El-Ganiny et al., 2008). Therefore, we investigated whether *ugmA* disruption also affected CFW resistance in *A. oryzae*. The growth of Δ*ugmA* was delayed on MM supplemented with 30 μg/ml CFW compared with that of the parental strain (Figure 3C), suggesting that *ugmA* disruption changes the balance of cell wall components, such as chitin and glucan, in *A. oryzae*.

**Figure 4:**
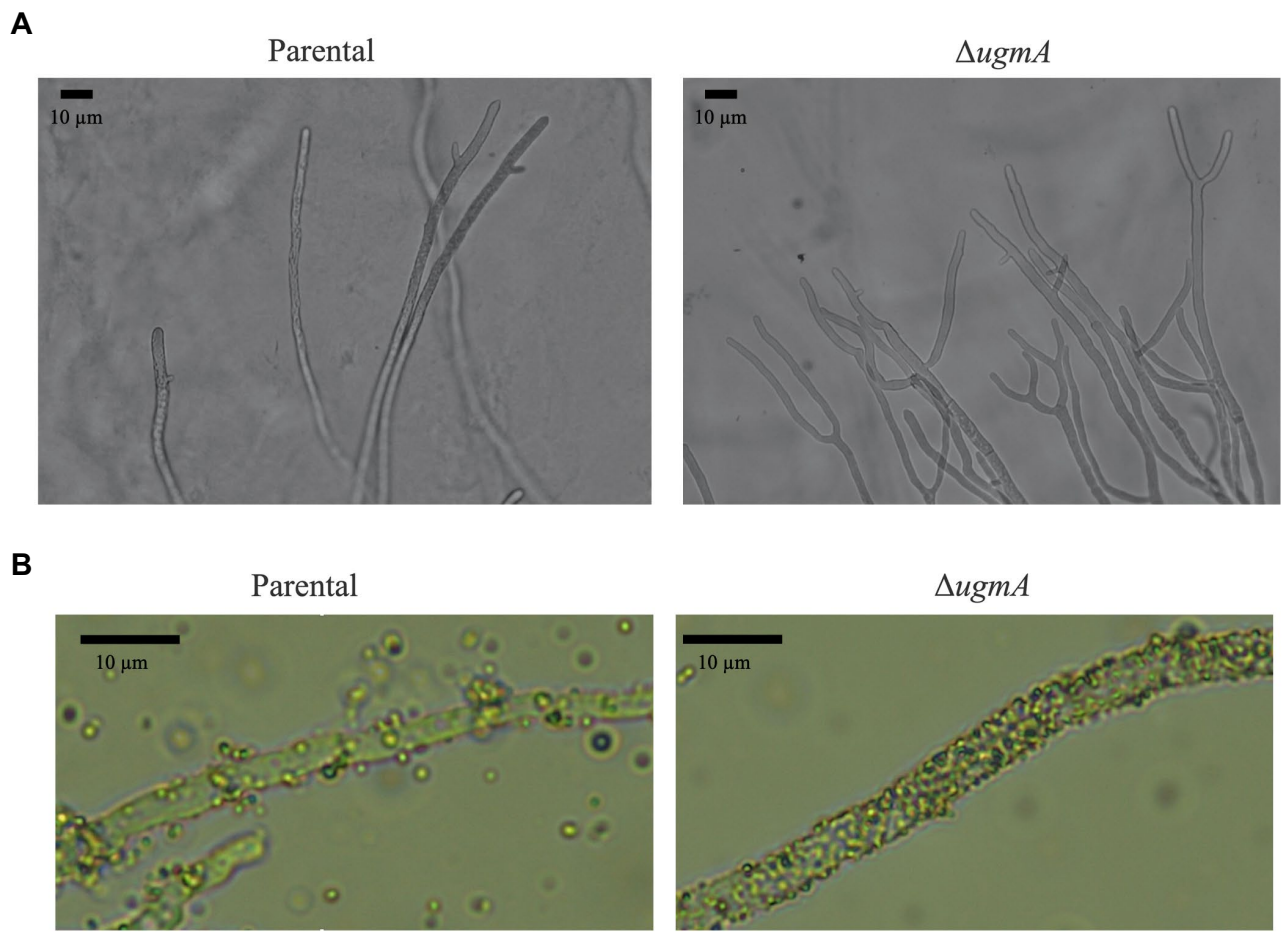
Morphology of the parental and Δ*ugmA* strains. **(A)** Morphology of the hyphae of the parental and Δ*ugmA* strains. **(B)** Hydrophobicity of the hyphae of the parental and Δ*ugmA* strains. Hydrophobicity was indicated by the adherence of latex beads to the hyphae.

It was previously reported that Δ*glfA* and ∆*gfsABC* strains of *A. fumigatus* exhibited increased hyphae branching (Lamarre et al., 2009; Chihara et al., 2020). Therefore, we examined whether abnormal mycelial branching also occurred in the Δ*ugmA* strain of *A. oryzae*. Branching structures at the hyphal tips were observed at a high frequency in the Δ*ugmA* strain (Figure 4A), indicating that the loss of Gal*f*-containing oligosaccharides increases abnormal mycelial branching. It has also been reported that the deletion of Gal*f*-containing oligosaccharides from cells increases cell surface hydrophobicity (Lamarre et al., 2009; Chihara et al., 2020). To confirm the increase in cell surface hydrophobicity of the Δ*ugmA* strain, we examined the level of attachment of latex beads to the mycelium in the Δ*ugmA* strain and found that the level of attachment was clearly increased (Figure 4B), suggesting that Gal*f-*containing oligosaccharides are involved in the cell surface hydrophobicity of *A. oryzae*.

### 3.4. Complementation test of *ugmA* expression in *Aspergillus nidulans* Δ*Anugm*A

**Figure 5:**
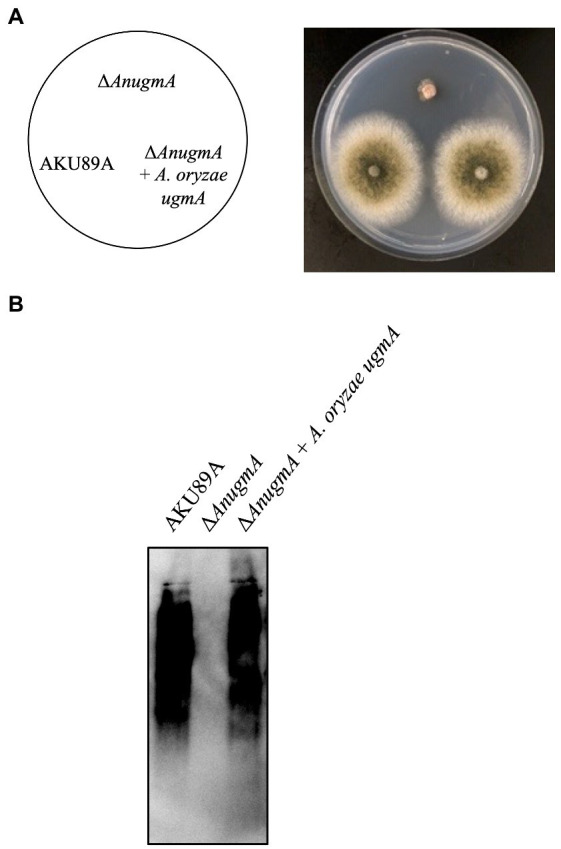
Expression of *Aspergillus oryzae ugmA* complements the *A. nidulans* Δ*AnugmA* phenotype. **(A)** Colonial morphology of AKU89A (control), Δ*AnugmA*, and Δ*AnugmA* + *A. oryzae ugmA* strains after culture on MM agar at 30°C for 4 days. Agar medium was inoculated with 1.0 × 10^4^ conidiospores. **(B)** Immunoblot analysis of galactomannoproteins using EB-A2. Lanes 1–3: 20 μg of galactomannoproteins extracted from AKU89A (Lane 1), Δ*AnugmA* (Lane 2), and Δ*AnugmA* + *A. oryzae ugmA* (Lane 3) were loaded.

To determine whether *A. oryzae ugmA* can complement the growth defect and Gal*f* antigen-lacking phenotype in *A. nidulans* Δ*AnugmA*, *ugmA* was expressed in *A. nidulans* Δ*AnugmA*. First, we evaluated whether the expression of *A. oryzae ugmA* could reverse the growth defects and aberrant conidial formation of *A. nidulans* Δ*ugmA*. Each conidium was inoculated on MM agar and cultivated at 37°C for 3 days. Expression of *A. oryzae ugmA* recovered the phenotype of *A. nidulans* Δ*AnugmA*, suggesting that *A. oryzae ugmA* complements the function of *A. nidulans* Δ*AnugmA* (Figure 5A). Next, we analyzed the presence of the Gal*f* antigen on the glycoprotein using EB-A2 antibody. Disruption of *AnugmA* results in the loss of Gal*f* antigens in glycoproteins in *A. nidulans* (Komachi et al., 2013), but expression of *A. oryzae ugmA* restored the Gal*f* antigen (Figure 5B). These results indicated that *A. oryzae ugmA* encodes a UDP-galactopyranose mutase.

### 3.5. Deficiency of galactofuranose residues on FTGM in Δ*ugmA* in the *A. oryzae* cell wall

**Figure 6:**
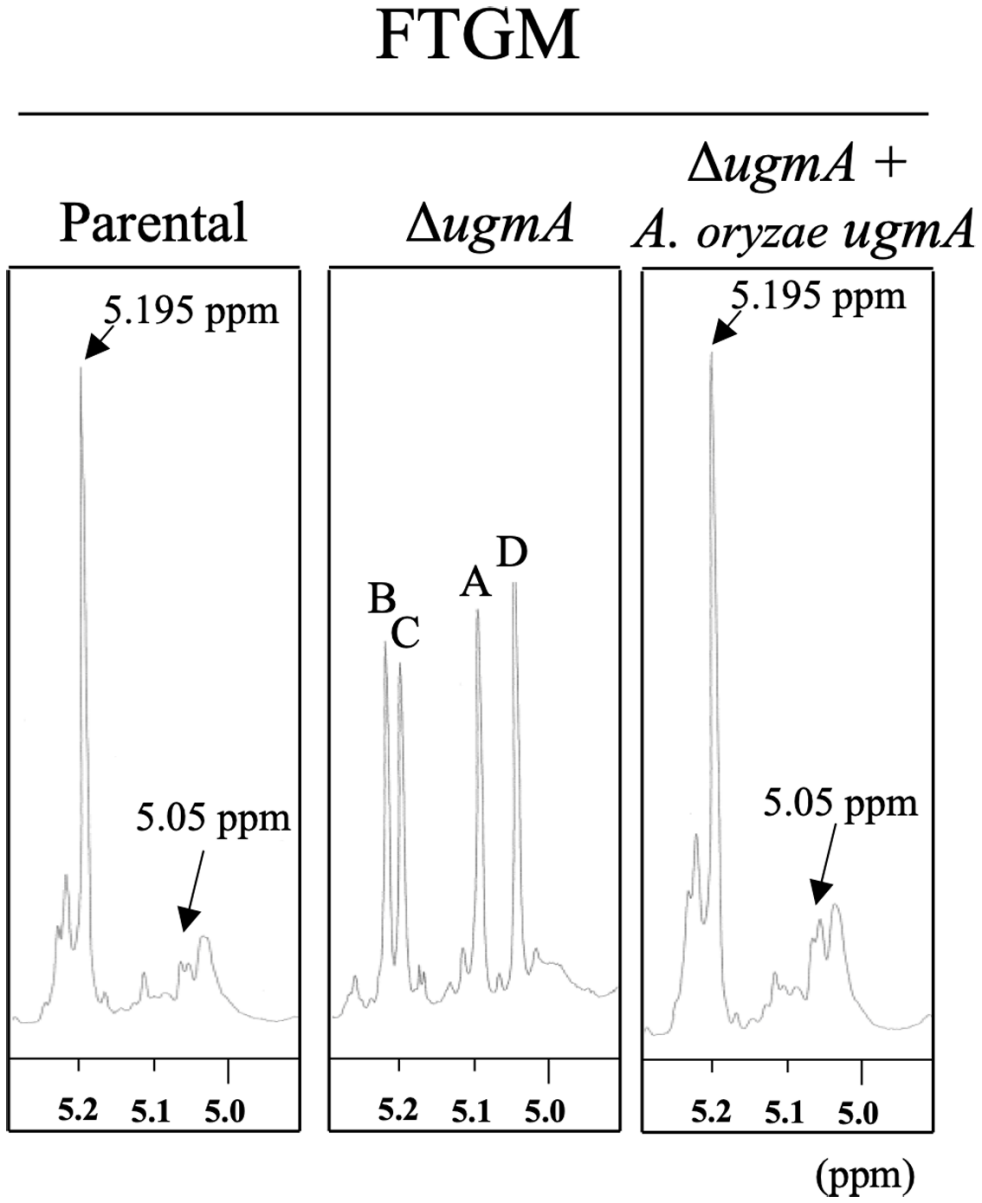
Proton nuclear magnetic resonance (^1^H-NMR) spectrometry of fungal-type galactomannan (FTGM) fraction from *Aspergillus oryzae*. The signal at 5.195 and 5.05 ppm of the ^1^H-NMR spectra is the H-1 signal of the C-1 position of the underlined Gal*f* residue in the β-Gal*f*-(1,5)-β-Gal*f*-(1,5)-β-Gal*f* and β-Gal*f*-(1,5)-β-Gal*f*-(1,6)-β-Gal*f* structures (Kudoh et al., 2015). Signals A (5.104 ppm), B (5.233 ppm), C (5.216 ppm), and D (5.054 ppm) from the ^1^ H-NMR spectra were derived from H-1 at the C-1 position of the underlined D-Mannose (Man) residues in the structures -(1,6)-α-Man-(1,2)-α-Man-(1,2)-α-Man-(1,2)-α-Man-(1,6)- **(A)**, −(1,6)-α-Man-(1,2)- α-Man-(1,2)- α-Man-(1,2)-α-Man-(1,6)- **(B)**, −(1,6)-α-Man-(1,2)-α-Man-(1,2)-α-Man-(1,2)-α-Man-(1,6)- **(C)**, and -(1,6)-α-Man-(1,2)-α-Man-(1,2)-α-Man-(1,2)-α-Man-(1,6)- **(D)**. The proton chemical shifts were referenced relative to internal acetone at δ 2.225 ppm.

To determine whether the galactofuranose-containing sugar chain is deficient in the ∆*ugmA* strain of *A. oryzae*, the galactofuranosyl residues were detected by ^1^H-NMR (Figure 6). The chemical shift signals at 5.195 and 5.05 ppm of the ^1^H-NMR spectra represented the H-1 signal of the underlined Gal*f* residue in the β-Gal*f*-(1,5)-β-Gal*f*-(1,5)-β-Gal and β-Gal*f*-(1,5)-β-Gal*f*-(1,6)-β-Gal*f* structures, respectively, according to previous studies on *A. fumigatus* (Shibata et al., 2009; Kudoh et al., 2015). Chemical shift signals at 5.195 and 5.05 ppm were detected in the GM fraction of the parental strain, consistent with the chemical shift signals of the β-(1,5)−/β-(1,6)-galactofuran side chain of FTGM in *A. fumigatus* (Figure 6). This result indicated the presence of the β-(1,5)−/β-(1,6)-galactofuran side chain structure in *A. oryzae*. In contrast, these signals were absent in the GM fraction of the ∆*ugmA* strain, indicating that the galactofuranose-containing sugar chain is deficient in the ∆*ugmA* strain of *A. oryzae* (Figure 6). In addition, four unique chemical shifts at 5.0–5.2 ppm (signals A–D), indicating the presence of core mannan backbone structures, emerged in the GM fraction of Δ*ugmA*, indicating the presence of a core mannan chain in FTGM (Kudoh et al., 2015; Onoue et al., 2018; Kadooka et al., 2022b). In the Δ*ugmA* + *ugmA* strain, the chemical shift indicating the presence of the α-core-mannan backbone was masked, and the chemical shifts indicating the galactofuran chain reappeared (Figure 6). These results clearly indicate that a structure consistent with the FTGM of *A. fumigatus* is also present in *A. oryzae*.

## 4. Discussion

It is important to analyze the substances produced by the microorganisms used in the production of fermented foods to ensure the foods’ safety. In this study, we focused on glycan structures and found that *A. oryzae* produces Gal*f*-containing glycans, such as galactomannoprotein and FTGM. Gal*f*-containing glycans are found in animal and plant pathogenic fungi such as *A. fumigatus* and *Fusarium* spp. (Takegawa et al., 1997; Tefsen et al., 2012; Oka, 2018; de Oliveira et al., 2019; Lee et al., 2019), and their relevance to the demonstration of virulence has attracted attention. Therefore, the Gal*f*-containing glycan of *A. oryzae* is likely a remnant of its phytopathogenic ancestor before its domestication. *A. oryzae* is commonly used in the production of Japanese fermented foods, such as miso, soy sauce, and sake, and Japanese people are likely to habitually consume large quantities of Gal*f* antigen. Although the positive and/or negative effects of ingesting Gal*f* antigens on the human body are unknown, these Japanese foods are considered healthy and may have immunostimulatory effects and/or improve intestinal flora (Kitagaki, 2021). Indeed, Nomura et al. (2021) reported that heat-killed *A. oryzae* spores and cell wall extracts from *A. oryzae* spores had a soothing effect on dextran sodium sulfate-induced colitis. It is hoped that the health benefits of habitual Gal*f* antigen ingestion will be reported in future studies.

Immunoblot analysis using EB-A2 revealed the presence of Gal*f*-containing glycoproteins in *koji* fungi. *A. oryzae* contained lower amounts of Gal*f*-containing glycoproteins than *A. nidulans* and *A. fumigatus*, whereas *A. luchuensis* and *A. kawachii* produced large amounts of Gal*f*-containing glycoproteins. Because it is known that *ugmA* deletion results in severe growth defects in *A. niger*, it is likely that Gal*f*-containing glycans, including glycoproteins, function as important cell wall components in *Aspergillus* section *Nigri* (Damveld et al., 2008; Park et al., 2016). Conversely, Gal*f*-containing glycoproteins tend to be less abundant in *A. oryzae* than in other filamentous fungi. Such differences of phenotype among *Aspergillus* species could indicate that the contributions of Gal*f*-containing glycoproteins to cell wall composition and normal mycelial formation differ among different *Aspergillus* species.

This study demonstrated that disruption of *ugmA* causes growth defects in *A. oryzae*. This inhibition of growth in ∆*ugmA* strains has been observed in other *Aspergillus* species and appears to be a common phenomenon (Damveld et al., 2008; El-Ganiny et al., 2008; Schmalhorst et al., 2008), thus indicating that Gal*f*-containing glycan structures play an important role in cell wall integrity in *A. oryzae*. In fact, Δ*ugmA* exhibited greater sensitivity to CFW, suggesting that the abundance of cell wall components, such as chitin and glucan, was altered in this strain. In *A. niger*, it has been reported that *ugmA* disruption increases the expression of various genes involved in the biosynthesis of α-glucan, β-glucan, and chitin (Park et al., 2016; Arentshorst et al., 2020). Although Gal*f*-containing glycans are not major components of the cell wall, they might be important in maintaining the balance between glucans and chitin in filamentous fungi.

**Figure 7:**
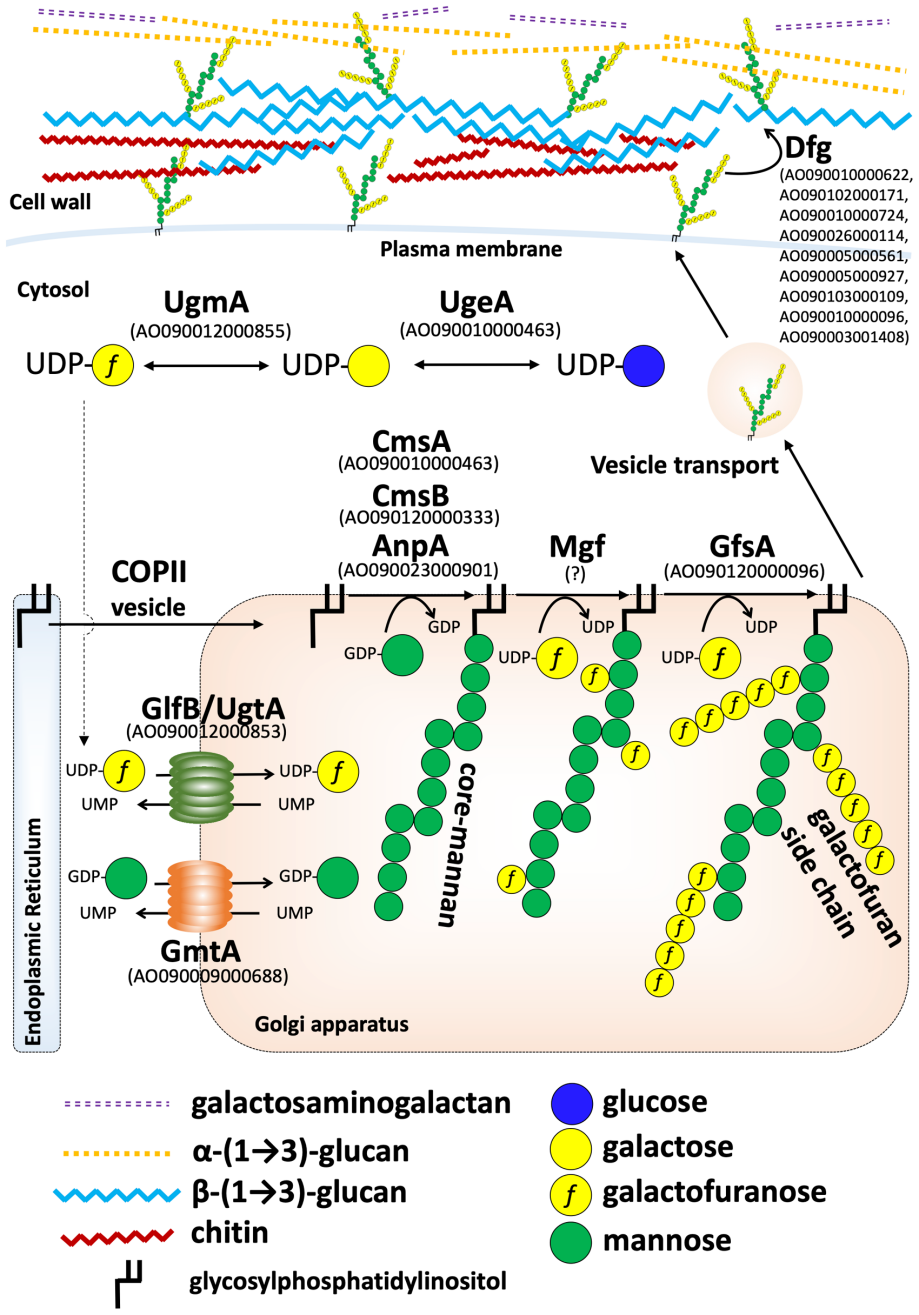
Predicted model of the fungal-type galactomannan (FTGM) biosynthetic pathway in *Aspergillus oryzae*. Homologs of each FTGM biosynthesis-related protein in *A. oryzae* were identified by BLASTP at NCBI using Uge5 (Afu5g10780/AFUB_058380), GlfB (Afu3g12700/AFUB_036470), GfsA (Afu6g02120/AFUB_096220), GmtA (Afu5g05740/AFUB_053290), CmsA (Afu5g02740/AFUB_051270), CmsB (Afu5g12160/AFUB_059750), and AnpA (Afu4g06870/AFUB_063940) of *A. fumigatus* as queries.

^1^H-NMR spectrometry suggested that a structure similar to the FTGM of *A. fumigatus* is present in the cell wall of *A. oryzae* (Figure 6). The signal indicating the presence of Gal*f* residues was completely lost in the *A. oryzae* ∆*ugmA* strain, indicating that UgmA is the only UDP-galactopyranose mutase in *A. oryzae* and that AoUgmB does not have the same enzymatic function as UDP-galactopyranose mutase (Figure 6). The level of Gal*f*-containing glycoproteins is lower in *A. oryzae* than in other filamentous fungi, suggesting that the growth defect of Δ*ugmA* is largely attributable to the loss of the galactofuran side chain of FTGM. The predicted biosynthesis map of FTGM in *A. oryzae* was illustrated based on previous studies (Figure 7). UDP-Gal*f* is synthesized from UDP-glucose *via* UDP-galactose by UgeA (AO090010000463) and UgmA in the cytosol (Damveld et al., 2008; El-Ganiny et al., 2008; Schmalhorst et al., 2008; El-Ganiny et al., 2010; Lee et al., 2014; Park et al., 2014). The *glfB*/*ugtA* homolog gene (AO090012000853), encoding a Golgi-localized UDP-Gal*f* transporter, is conserved adjacent to *ugmA* in the *A. oryzae* genome, as reported for *A. fumigatus* (Engel et al., 2009; Afroz et al., 2011; Park et al., 2015). Unlike other *Aspergillus* spp., in *A. oryzae*, only *gfsA* (AO090120000096) is present in the genome as a *gfsA/B/C* homolog encoding β-(1,5)-galactofuranosyltransferase (Komachi et al., 2013; Katafuchi et al., 2017; Chihara et al., 2020). Therefore, it is likely that only *gfsA* is involved in the biosynthesis of the β-(1,5)-galactofuran side chain of FTGM in *A. oryzae*. AO090009000688 is conserved as a homolog of the Golgi-localized GDP-Man transporter gene, *gmtA* (Jackson-Hayes et al., 2008; Engel et al., 2012). In addition, *cmsA* (AO090010000463) and *cmsB* (AO090120000333), encoding α-(1,2)-mannosyltransferase, and *anpA* (AO090023000901), encoding α-(1,6)-mannosyltransferase, which are involved in the biosynthesis of the FTGM core mannan backbone, are conserved in the *A. oryzae* genome (Onoue et al., 2018; Kadooka et al., 2022b). Although the enzymes that add Gal*f* to the core mannan backbone (α-mannoside β-galactofuranosyltransferase: Mgf) have not yet been identified in filamentous fungi, it can be inferred that the aforementioned genes are essential for the biosynthesis of FTGM in *A. oryzae*. Recent studies have postulated that FTGM is transported to the cell surface *via* the GPI-anchor and then cross-linked to β-glucan by Dfg proteins, which are mannosidases belonging to the GH76 family (Muszkieta et al., 2019; Fontaine and Latgé, 2020; Vogt et al., 2020). These FTGM-conjugated β-glucan structures are thought to play an important role in normal mycelial elongation in *A. oryzae*.

Previous studies have shown that the polysaccharides that compose the *A. oryzae* cell wall include β-(1,3)-glucan, α-(1,3)-glucan, chitin, and galactosaminogalactan (Müller et al., 2003; Zhang et al., 2017; Miyazawa et al., 2019, Figure 7). Through an analysis of *ugmA*, we revealed that *A. oryzae* cell walls also contain FTGM structures and galactomannoproteins. Our findings provide novel insights into the structure of the *A. oryzae* cell wall and the health-promoting effects of fermented foods made from the yellow *koji* fungus in Japan.

## Data availability statement

The original contributions presented in the study are included in the article/Supplementary material, further inquiries can be directed to the corresponding author/s.

## Author contributions

CK and TO designed and performed the experiments. YT performed the nuclear magnetic response analysis. DH performed the evolutionary phylogenetic analysis. CK, YT, and TO analyzed and interpreted the data. JM contributed materials. MG and JM participated in discussion of the study. TO planned and designed the project. CK and TO wrote the manuscript. All authors contributed to the article and approved the submitted version.

## Funding

This work was supported in part by Grant-in-Aid for Scientific Research (C) from the Japan Society for the Promotion of Science (JSPS KAKENHI; 18K05418 to TO, 21K05373 to TO, 22K06600 to YT, and 19K05835 to DH) and by Grant-in-Aid for Early-Career Scientists from Japan Society for the Promotion of Science (JSPS KAKENHI; 22K14817 to CK and 20K15997 to YT).

## Conflict of interest

The authors declare that the research was conducted in the absence of any commercial or financial relationships that could be construed as a potential conflict of interest.

## Publisher’s note

All claims expressed in this article are solely those of the authors and do not necessarily represent those of their affiliated organizations, or those of the publisher, the editors and the reviewers. Any product that may be evaluated in this article, or claim that may be made by its manufacturer, is not guaranteed or endorsed by the publisher.

## Footnotes

^1^ https://www.ncbi.nlm.nih.gov/
