# Supplementary material for: Identification of galactofuranose antigens such as galactomannoproteins and fungal-type galactomannan from the yellow koji fungus (Aspergillus oryzae)
Source: Front Microbiol. 2023 Feb 6;14:1110996. doi: 10.3389/fmicb.2023.1110996 (PMC9939772; doi:10.3389/fmicb.2023.1110996)
Supplement: Supplementary file 1 [file Data_Sheet_1.PDF]

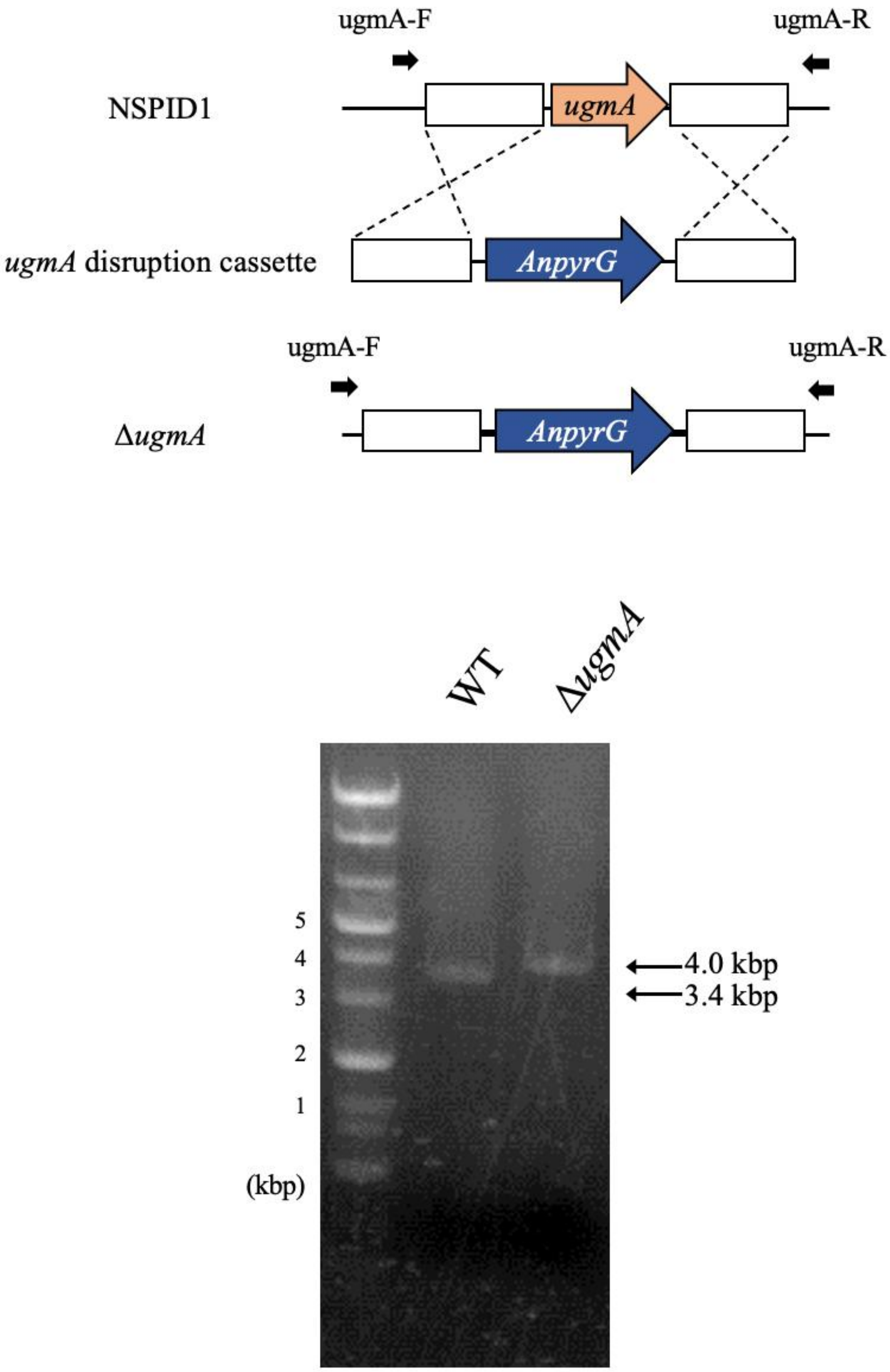

Supplementary Figure 1. Construction of the Δ*ugmA* strain. The results of electrophoretic analyses of the PCR products are presented in the bottom panel.

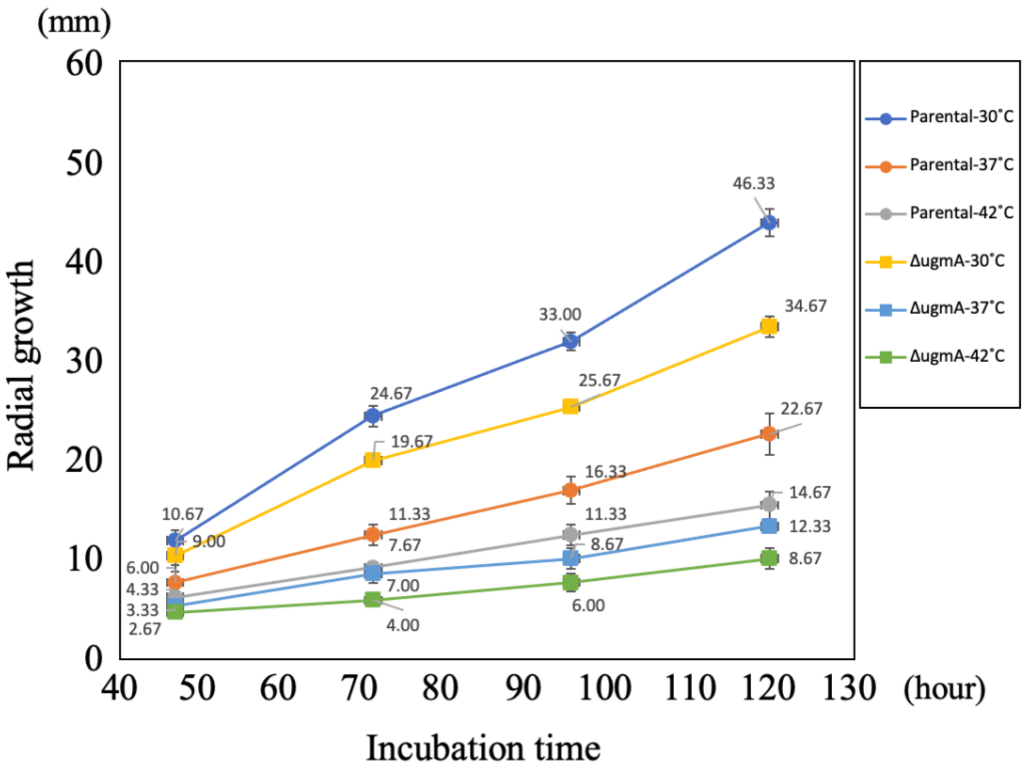

Supplementary Figure 2. Radial growth of the parental (NSPlD1) and Δ*ugmA* strains at various temperatures. Over 120 h, the colony diameter from the point of inoculation was measured every 24 h.

Table S1. Strains used in this study

Strains	Genotype	Source
Aspergillus oryzae		
RIB40	wild-type	Obtained from RIB
NSPlD1	*niaD* - *sC* - *adeA* - *argB* ::*adeA* - *ligD* ::*argB* *pyrG* ::*adeA*	Maruyama and Kitamoto. 2008
Δ*ugmA*	*niaD* - *sC* - *adeA* - *argB* ::*adeA* - *ligD* ::*argB* *pyrG* ::*adeA* *glfA* ::*pyrG*	This study
Δ*ugmA* + *A. oryzae ugmA*	*niaD* - *sC* - *adeA* - *argB* ::*adeA* - *ligD* ::*argB* *pyrG* ::*adeA* *glfA* ::*pyrG* pPTR-II-ugmA	This study
Aspergillus nidulans		
A26	*biA1*	Obtained from FGSC
AKU89A	*biA1* *argB2* ::*argB* *akuB* ::*AUR* $^{+}$	Komachi et al. 2013
Δ*AngmA*	*biA1* *argB2* *akuB* ::*AUR* $^{+}$ *AnugmA* ::*argB*	Komachi et al. 2013
Δ*AnugmA* + *A. oryzae ugmA*	*biA1* *argB2* *akuB* ::*AUR* $^{+}$ *AnugmA* ::*argB* pPTR-II-ugmA	This study
Other fungi		
Aspergillus kawachii IFO4308	wild-type	Obtained from IFO
Aspergillus luchuensis	alias: *A. awamori* var. *kawachi* , wild-type	Oka et al. 2005
Aspergillus fumigatus A1151	$pyrG^{AF}$::Delta KU80	Obtained from FGSC

Table S2. Primers used in this study

Name	Sequence (5'-3')	Source
pHSG396-F	CGAGGGGTCGACTCTAGAGG	Kadooka et al. 2022
pHSG396-R	TACCCCATCGATGGGGGATC	Kadooka et al. 2022
glfA-1	GCTAACCACAGCACAGGCA	This study
glfA-2	AGAGTCGACCCCTCGAGAAGGAGAGAGTGCTGCAG	This study
glfA-3	CCCATCGATGGGGTAGGCTGCGCGATCACTATAGA	This study
glfA-4	CGGTTTCGATTGGATGATGCG	This study
glfA-F	ATGGCAAGAACTACCCCGG	This study
glfA-R	ACTTACGCGTCCTACCCG	This study
pPTR-II-glfA-IF-F	CTCTAGAGGATCCCCTGCAATGGCAAGAACTACCC	This study
pPTR-II-glfA-IF-R	TCGAGCTCGGTACCCTTTGACATTGGAGGATCCCC	This study
